# Enhanced thioredoxin, glutathione and Nrf2 antioxidant systems by safflower extract and aceglutamide attenuate cerebral ischaemia/reperfusion injury

**DOI:** 10.1111/jcmm.15099

**Published:** 2020-04-07

**Authors:** Jingjing Zhang, Rui Zhou, Changpei Xiang, Fangfang Fan, Jinhuan Gao, Yi Zhang, Shihuan Tang, Haiyu Xu, Hongjun Yang

**Affiliations:** ^1^ Institute of Chinese Materia Medica China Academy of Chinese Medical Sciences Beijing China

**Keywords:** aceglutamide, glutathione, Nrf2, oxidative stress, stroke, thioredoxin

## Abstract

A large number of reactive oxygen species (ROS) aggravate cerebral damage after ischaemia/reperfusion (I/R). Glutathione (GSH), thioredoxin (Trx) and nuclear factor (erythroid‐derived 2)‐like 2 (Nrf2) represent three major antioxidant systems and play vital roles in affecting each other in eliminating ROS. Identification of drugs targeting triple antioxidant systems simultaneously is vital for inhibiting oxidative damage after cerebral I/R. This study investigated the protective effect of safflower extract and aceglutamide (SAAG) against cerebral I/R injury through modulating multiple antioxidant systems of GSH, Trx and Nrf2 and identified each role of its component acegluatminde (AG) and safflower extract (SA) on these systems. Safflower extract and aceglutamide and its two components decreased neurological deficit scores, infarction rate, apoptosis and oxidative damage after cerebral I/R while enhanced cell viability, decreased reactive oxygen species and nitric oxide level in H_2_O_2_‐induced PC12 cell model. Importantly, compared to its two components, SAAG demonstrated more effective enhancement of GSH, Nrf2 and Trx systems and a better protection against cerebral I/R injury. The enhanced antioxidant systems prevented ASK1 activation and suppressed subsequent p38 and JNK cascade‐mediated apoptosis. Moreover, inhibition of Trx and Nrf2 systems by auranofin and ML385 abolished SAAG‐mediated protection, respectively. Thus, enhanced triple systems by SAAG played a better protective role than those by SA or AG via inhibition of ASK1 cascades. This research provided evidence for the necessity of combination drugs from the perspective of multiple antioxidant systems. Furthermore, it also offers references for the study of combination drugs and inspires novel treatments for ischaemic stroke.

## 1 INTRODUCTION

Ischaemic stroke, which is caused by a sudden cessation of blood flow by a thrombus or embolism obstruction in the brain, accounts for approximately 80% of all stroke and is a leading cause of disability, morbidity and mortality worldwide.^1^ Recombinant tissue plasminogen activator (rtPA), the only FDA‐approved medication for ischaemic stroke, has many limitations due to narrow therapeutic time window and serious side effects, such as potential haemorrhagic risks and limited efficacy.^2^ According to the statistics, less than 8% of patients with acute ischaemic stroke have received rtPA treatment.^3^ However, subsequent reperfusion after cerebral ischaemia causes more serious damage than the occlusion itself.^4^, ^5^ Thus, the identification of effective therapeutic strategies for ischaemic stroke is imperative.

Notably, the brain is highly sensitive and vulnerable to oxidative damage due to its high oxygen demand and very limited antioxidant capacity.^6^ Once cerebral ischaemia/reperfusion (I/R) is initiated, a large number of reactive oxygen species (ROS) are produced and attack various cellular macromolecules, resulting in DNA double‐strand breakage, protein oxidation, lipid peroxidation and ultimately cell death.^7^, ^8^, ^9^ Antioxidant strategies aimed at exhausting excess free radicals have achieved effective protection against cerebral I/R injury.^10^ Thioredoxin (Trx), glutathione (GSH) and Nrf2 systems are three major antioxidant systems responsible for removing overproduced free radicals. Glutathione, a thiol‐based antioxidant present in millimolar concentrations in the brain, plays a vital role in modulating redox homoeostasis.^11^, ^12^ GSH depletion is involved in multiple neurological diseases, including cerebral ischaemic stroke,^13^, ^14^, ^15^ whereas the administration of GSH reduces the infarct volume and ameliorates brain damage.^16^, ^17^, ^18^ Moreover, Trx is another important antioxidant system that catalyses thiol‐disulphide oxidoreductions in DNA synthesis, defends against oxidative injury and inhibits apoptosis. In particular, Trx binds to apoptosis signal‐regulating kinase 1 (ASK1) and inhibits subsequent apoptosis in cerebral I/R injury.^18^, ^19^ In addition, Nrf2 is a central regulator that modulates redox homoeostasis by binding to its antioxidant response elements (AREs) in the promoter regions of target genes in response to oxidative stress.^20^ In cerebral I/R injury, the functions of multiple antioxidant systems are seriously damaged, and the brain is vulnerable to the excess oxygen free radicals that aggravate cerebral damage.

A growing number of evidences suggest that GSH, Nrf2 and Trx antioxidant systems can affect each other and play vital roles in removing overproduced ROS.^21^ For example, the dual inhibition of GSH and Trx caused cancer cell death but led to the activation of Nrf2 system, which decreased the therapeutic effect.^21^ Additionally, targeting the three antioxidant systems of Trx, GSH and Nrf2 simultaneously is more effective at eliminating cancer than targeting only two of these systems.^22^ What's more, Nrf2 activation stimulates the expression of a large number of antioxidant genes, including GCLM and GCLC (key enzymes required for GSH synthesis) and Trx1 (a key component of the Trx system). Nrf2 alleviates cerebral I/R damage by regulating the Trx/TXNIP complex.^23^, ^24^ Additionally, GSH maintains the reduced form of Trx1 in the absence of TrxR1 activity.^25^ All these researches indicate these three systems affect each other and can work together to remove ROS. Thus, we hypothesized that an enhancement of the Trx, GSH and Nrf2 systems would effectively alleviate oxidative damage after cerebral I/R injury. Whereas, the effect of simultaneous enhancement of the Trx, GSH and Nrf2 systems on cerebral I/R has been rarely investigated. Thus, identification of drugs targeting triple antioxidant systems simultaneously is vital for the inhibition of oxidative damage after cerebral I/R.

**Figure 1:**
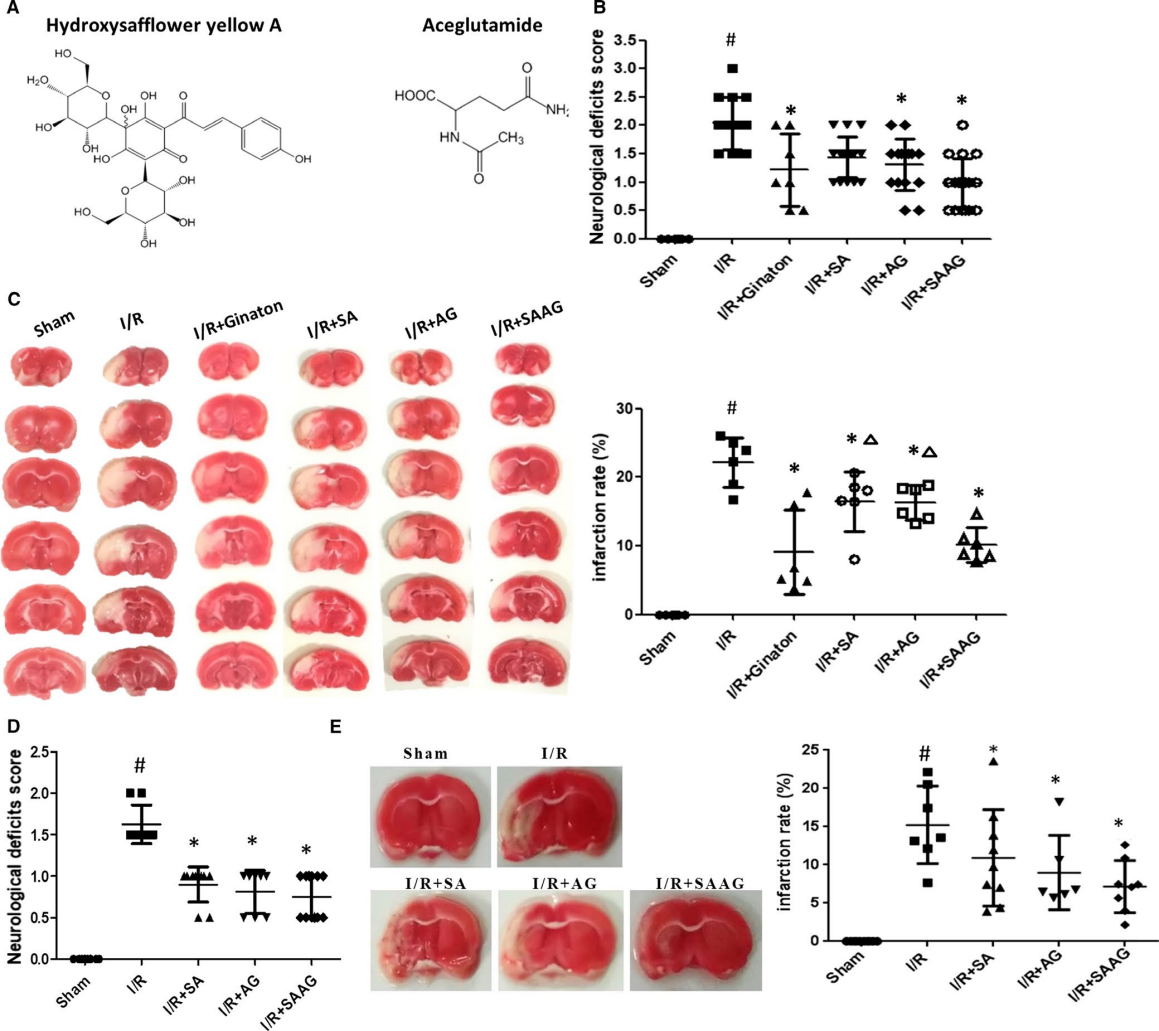
SAAG, SA and AG protected against cerebral I/R injury at day 2 and day 12. A, The chemical structures of hydroxysafflower yellow A (the main ingredient of SA) and AG; B, Neurological deficient scores at day 2 (n = 7 for I/R + Ginaton and n = 14‐18 for other groups); C, Representative TTC staining images with the quantified infarction ratio using imageJ at day 2 (n = 6); D, Neurological deficient scores at day 12 (n = 8‐12); E, Representative TTC staining images with the quantified infarction rate using imageJ at day 12 (n = 6‐8); According to the instruction of SAAG, every millilitre of SAAG contained 30 mg AG and 0.5 g SA, thus 1.25 g/kg of SA and 75 mg/kg of AG were also evaluate to investigate the role of each composition of SAAG on the protection against cerebral I/R injury, respectively. ^#^ *P* < .05 compared with the sham group, *P < .05 compared with the I/R group, and ^∆^ *P* < .05 compared with the I/R + SAAG group

Aceglutamide (AG), clinically used for brain trauma, mental retardation, memory loss after cerebrovascular disease, coma induced by various causes, has been reported to promote the GSH system and benefits the recovery of ischaemic stroke.^26^, ^27^ Safflower (*Carthamus tinctorius* L.) can significantly enhance Nrf2 and has demonstrated an obvious cerebral protective effect after I/R with its main effective component, hydroxysafflower yellow A ^28^, ^29^ (Figure 1A). These data indicating the combination of SA and AG may have an important effect in enhancing Nrf2, Trx and GSH systems. Notably, combination drugs represented by safflower extract and aceglutamide (SAAG) are a standardized medicine product recorded in the Chinese Pharmacopeia, which is composed of SA and AG, are widely used for various cerebral diseases, such as clinical cerebral insufficient blood supply and cerebral thrombosis and embolism, and have shown an obvious protective effect against cerebral I/R injury.^30^, ^31^ Our previous research indicated SAAG had an obvious protective effect against cerebral ischaemic stroke^32^; however, the mechanism of SAAG remains unclear, especially on regulating multiple antioxidant systems. What's more, the role of the two components in promoting triple antioxidant system needs further investigation. In this study, the protective effect of SAAG against cerebral I/R by modulating triple antioxidant systems of GSH, Trx and Nrf2 was investigated and the role of each component on these systems was identified.

## 2 MATERIALS AND METHODS

### 2.1 Animals and materials

Adult Sprague‐Dawley rats (male, 280‐320 g) were obtained from the Experimental Animal Center of Peking University Health Science Center, Beijing, China [(certificate no. SCXK (Jing) 2009‐0017)]. All animal experiments were performed strictly in accordance with the guidelines and regulations of the Committee on Animal Care and Use of the Institute of Chinese Materia Medica, China Academy of Chinese Medical Sciences. All rats were allowed free access to food and water and were housed at an ambient temperature on a 12‐hour light/dark cycle. Safflower extract and aceglutamide injection (SAAG, batch number: 20 170 402) were offered by Tonghua Guhong Pharmaceutical Co., Ltd. (Chinese medicine character: H22026582) as a combination of AG and SA. Details information about drugs and antibodies was described in Appendix S1.

### 2.2 Middle cerebral artery occlusion (MCAO) surgery and drug administration

Rats were anaesthetized with sodium pentobarbital (45 mg/kg, intraperitoneal injection, ip) and were subjected to MCAO surgery for occlusion for 90 minutes and then followed by reperfusion for 2 and 12 days, respectively. Administration of 2.5 mL/kg SAAG, 1.25 g/kg SA and 75 mg/kg AG were administrated by intraperitoneal injection every day. All of the groups were derived from a random number table. Details information about drugs and antibodies was described in Appendix S1.

### 2.3 Intracerebroventricular injection

Briefly, auranofin (10 μL, 10 mmol/L) was administered 30 minutes after MCAO‐induced ischaemia and was injected into the lateral ventricle on the side of the operation within 10 minutes at a speed of 1 μL/min. Safflower extract and aceglutamide (2.5 mL/kg, ip) was administered to the appropriate group at the same time. After 24 hours, auranofin (10 μL, 10 mmol/L) was again administered by an injection (ip) immediately after administration of SAAG (ip). Details about the surgery were described in Appendix S1.

### 2.4 Neurological deficit scores and TTC staining

The Longa 5 methods were used to evaluate the neurological deficit scores.^33^ The Longa 5 method was used with a slight modification, and the situation between two levels of score was also scored. For example, the rats were rated as 1.5, when situation of was more serious than the score of 1, but lighter than the score of 2. And 2,3,5‐Triphenyltetrazolium chloride (TTC, 2% w/v, Sigma) staining was used to stain the 2‐mm‐thick cerebral sections at 37°C for 30 minutes. Photos were taken for ImageJ analysis of the infarction rate. The infarction rate was calculated as follows: infarction rate = the infarcted cerebral area/the overall cerebral area.

### 2.5 Biochemical analysis of Nitric Oxide level, GSH level, MDA content and activities of SOD, GSH‐Px, TrxR and caspase‐3

Kits for detecting nitric oxide (NO, A013‐2‐1), superoxide dismutase (T‐SOD, A001‐3), malondialdehyde (MDA, A003‐1), GSH (A006), thioredoxin reductase (TrxR, A119), glutathione peroxidase (GSH‐Px, A005), glutathione reductase (GR, A062) and caspase‐3 activity (A007) were obtained from Nanjing Jiancheng Bioengineering Institute and were performed according to the respective protocols with a microplate reader (Molecular Devices, USA). The caspase‐3 activity was determined according to the manuscript instructions. Briefly, 50 μg of total protein was loaded and incubated with 25 μL Ac‐DEVD‐pNA at 37°C for 4 hours before being measured with a microplate reader (Molecular Devices, USA) at 405 nm.

### 2.6 Western blotting and immunofluorescence staining

The total protein of brain tissue was harvested and quantified with a Beyotime Protein Assay Kit, which was used for the following Western blotting. As for detecting Nrf2 in cell samples, nucleus extract of protein lysate was used. After denaturation, the proteins were separated by electrophoresis on polyacrylamide gels, followed by transfer of the proteins to polyvinylidene difluoride membranes, which were blocked with bovine serum albumin (BSA). Then, the membranes were incubated with primary antibodies, followed by incubation with HRP‐conjugated secondary antibody (Jackson 111‐035‐003). Using an enhanced chemiluminescence plus detection system (Pierce Biotechnology), the protein signals on the membranes were quantified by scanning densitometry with image analysis software (Science Lab 2005 Image Gauge).

As for immunofluorescence (IF) staining, sections of brain tissue with 5‐μm thickness were permeabilized with 0.5% Triton X‐100, followed by blocking with 5% BSA. After primary antibody incubation overnight at 4°C, the sections were incubated with Alexa Fluor 488‐ and Alexa Fluor 647‐conjugated secondary antibodies with DAPI (Sigma) for the nuclei staining. The images were examined with an LSM‐880 confocal microscope (Carl Zeiss).

### 2.7 PC12 cell culture and drug administration

Differentiated PC12 cells were purchased from Peking Union Medical College. The cells were cultured in DMEM/F12 (Gibco) with 10%FBS (Gibco), 100 U/mL penicillin and 100 U/mL streptomycin at 37°C in a humidified incubator with 5% CO_2._ Briefly, cells were incubated with drugs for 24 hours to investigate the safe concentration. To test the therapeutic effect, PC12 cells were pre‐treated with or without SAAG for 24 hours and then replaced by H_2_O_2_ for 1 hour. The cell viability was measured with CCK‐8 kits, which was obtained from Dongren Chemical Technology Co., Ltd.

### 2.8 Nrf2 activation and ROS measurement

The nuclear protein was harvested with Nuclear Extraction Kit (ab113474, Abcam) and detected with BCA kits (Thermo Fisher). The Nrf2 activation was detected by using the nuclear protein with Nrf2 Transcription Factor Assay Kit (Colorimetric) (ab207223, Abcam) with a microplate reader (Molecular Devices) at 450 nm following the kit's protocol.

Reactive oxygen species Assay Kit (Beyotime, S0033) was used to detect cellular ROS level after cell pre‐treatment with drugs and H_2_O_2_. After that, DCFH‐DA was added to incubate with cells for 20 minutes at 37°C. Reactive oxygen species levels were detected at 525 nm with a microplate reader (Molecular Devices).

### 2.9 Statistical analysis

The data are presented as the means ± standard deviations and analysed with SPSS software. Student *t* test was used to analyse data between Sham and I/R group while the results of I/R, I/R + AG, I/R + SA and I/R + SAAG was analysed by using One‐way ANOVA with Tukey post hoc test. Neurological deficit scores in Figures 1B and 6A were analysed using Kruskal‐Wallis test and Mann‐Whitney *U* test, respectively. *P* < .05 was supposed to have statistical significance.

## 3 RESULTS

### 3.1 SAAG, SA and AG showed an obvious protective effect against I/R injury

Our previous research identified 2.5 mL/kg SAAG had an obvious protective effect against cerebral ischaemic I/R^32^; however, the mechanism of SAAG remained unclear, especially on regulating multiple antioxidant systems. Safflower extract and aceglutamide demonstrated a good protective effect when compared to I/R group as evidenced by the remarkable decrease in neurological scores and infarction rate (*P* < .05, Figure 1). As for its two compositions, AG significantly reduced infarction rate and Longa 5 neurological scores (*P* < .05) while SA only decreased infarction rate (*P* < .05). Importantly, SAAG demonstrated a better protective effect than either AG or SA as evidenced by the reduced infarction rate (*P* < .05) and Longa 5 neurological scores (not significant). At day 12, SAAG, SA and AG significantly reduced neurological deficient scores and remarkably decreased infarction ratio and (*P* < .05, Figure 1D,E). These data indicated that SAAG and its two compositions SA and AG protected against cerebral I/R injury at day 12.

### 3.2 SAAG, SA and AG enhanced the multiple antioxidant systems of Trx, GSH and Nrf2

**Figure 2:**
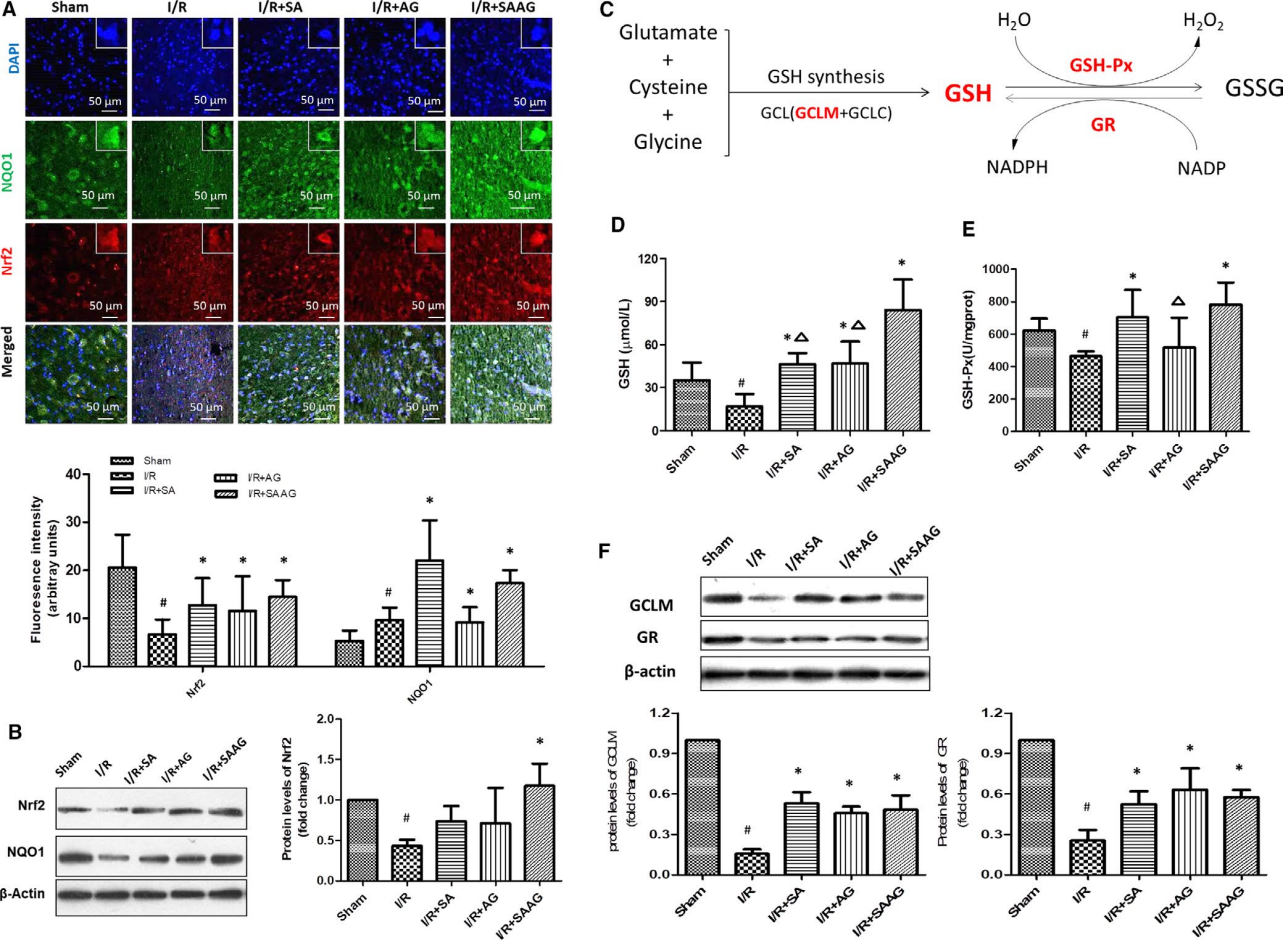
SAAG, SA and AG enhanced the Nrf2 and GSH systems. A, Immunofluorescence staining for Nrf2 (red), NQO1 (green) and their quantitation of fluorescence intensity (arbitrary units, n = 3‐5 animals per group); nuclei (blue), scale bar: 50 μm. B, Western blotting of Nrf2 and NQO1 and the quantitation of Nrf2 levels (n = 3). C, Schematic diagram illustrating GSH systems which includes GSH, GSH‐Px, GR and so on (the molecules labelled red were detected molecules); D, GSH levels (n = 6); E, GSH‐Px activity (n = 6). F, Western blotting of GCLM and GR and their quantitation (n = 3); ^#^ *P* < .05 compared with the sham group, *P < .05 compared with the I/R group, and ^∆^ *P* < .05 compared with the I/R + SAAG group

As indicated in Figure 2A, immunofluorescence staining results indicated SAAG, SA and AG obviously increased Nrf2 and NQO1 expression levels (*P* < .05, compared to I/R). The Western blotting results demonstrated that SAAG rather than its two components, significantly increased Nrf2 protein levels (*P* < .05, Figure 2B). As for GSH systems, GSH level, GSH‐Px activity, glutamate‐cysteine ligase regulatory subunit (GCLM, a rate‐limiting enzyme for GSH synthesis) and glutathione reductase (GR, a key enzyme that reduces GSSG to GSH) were detected accordingly. The decreased GSH levels in I/R group were significantly increased by SA, AG and SAAG (*P* < .05). Notably, the SAAG treatment increased the GSH level to a greater extent than either the SA or AG treatment (*P* < .05). GSH‐Px activity was remarkably increased by SAAG and SA treatment (*P* < .05 compared to I/R, Figure 2E). Additionally, SAAG, SA and AG significantly elevated GCLM and GR levels (*P* < .05 compared to I/R, Figure 2F). Thus, SAAG and its two components exerted a good effect on enhancing GSH system and Nrf2 system.

**Figure 3:**
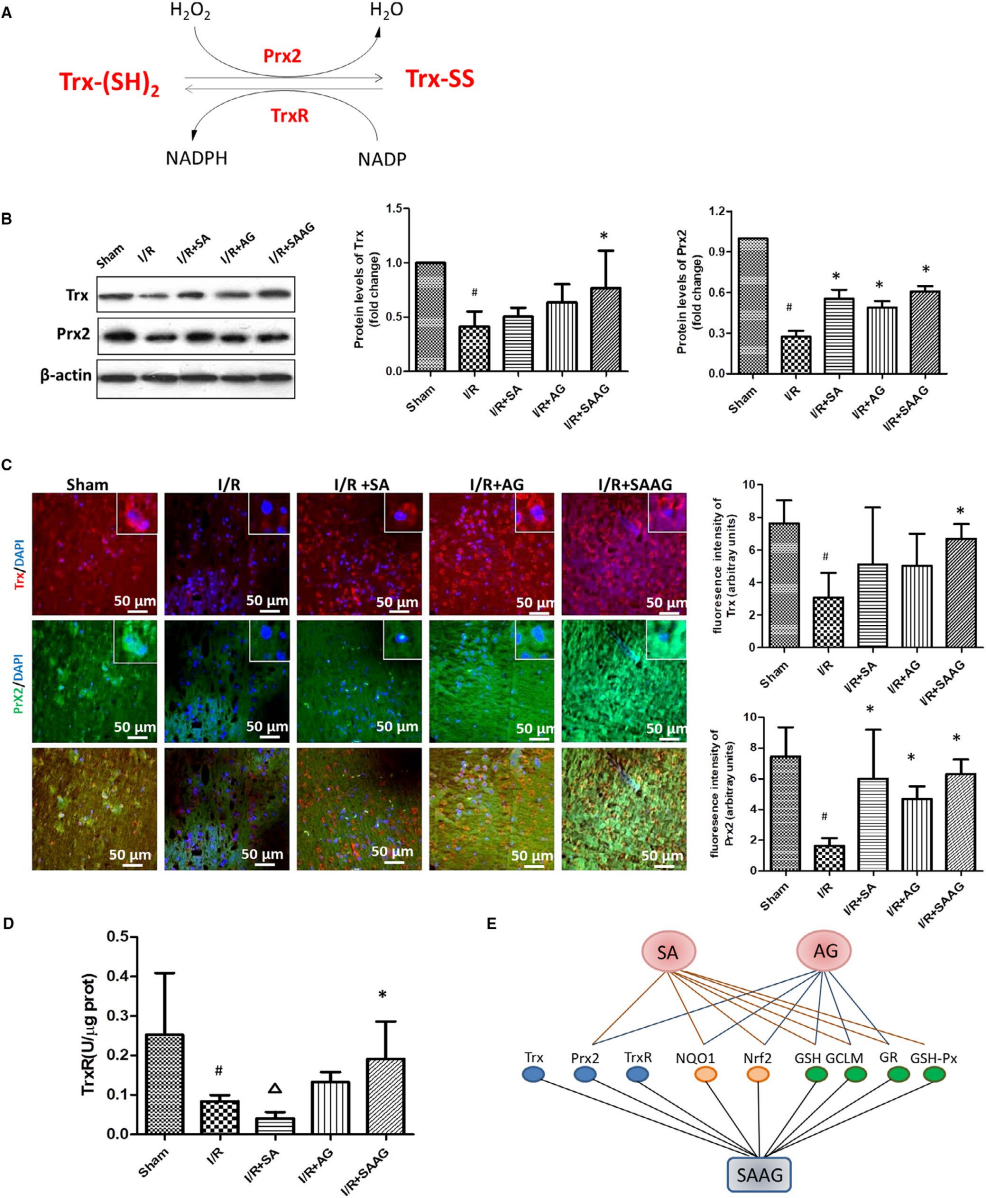
SAAG and its two components, SA and AG, enhanced the Trx system and a schematic diagram summarizing the effect of SAAG and its two components on the functions of the GSH, Nrf2 and GSH systems. A, Schematic diagram illustrating the Trx systems which includes Trx, Prxs, TrxR and NADPH (the molecules labelled red were detected molecules); B, Western blotting of Trx and Prx2 and their quantitation (n = 3). C, Immunofluorescence staining for Prx2 (green), Trx (red) and their quantitation of fluorescence intensity (arbitrary units, n = 3‐5 animals per group); nuclei (blue), scale bar: 50 μm. D, TrxR activity (n = 6); ^#^ *P* < .05 compared with the sham group, *P < .05 compared with the I/R group, and ^∆^ *P* < .05 compared with the I/R + SAAG group. E, Schematic diagram summarizing the effects of SAAG, SA and AG on the functions of the GSH, Nrf2 and GSH systems. Trx systems include Trx, Prx2 and TrxR, as indicated by blue dots; Nrf2 systems include NQO1 and Nrf2, as indicated by orange dots, while GSH systems include GSH, GCLM and GR, as indicated by green dots. The line between SAAG, SA and AG and the biomolecules of these systems represents the significance that was observed by experiments when compared with the I/R group

Safflower extract and aceglutamide rather than its two components (SA or AG) obviously enhanced the decreased Trx levels in I/R (*P* < .05), meanwhile the decreased Prx2 level in I/R group was remarkably elevated by SAAG, SA and AG treatment (*P* < .05, Figure 3B,C). Compared with SA or AG treatment, SAAG treatment had a better effect on enhancing Trx (*P* < .05). The repressed TrxR activity in I/R group was obviously enhanced by SAAG (*P* < .05, Figure 3D), but not by SA or AG. In conclusion, SAAG demonstrated a better improved effect on the functions of these systems than either SA or AG.

### 3.3 SAAG, SA and AG inhibited the ASK1‐dependent JNK/p38 signalling pathway

**Figure 4:**
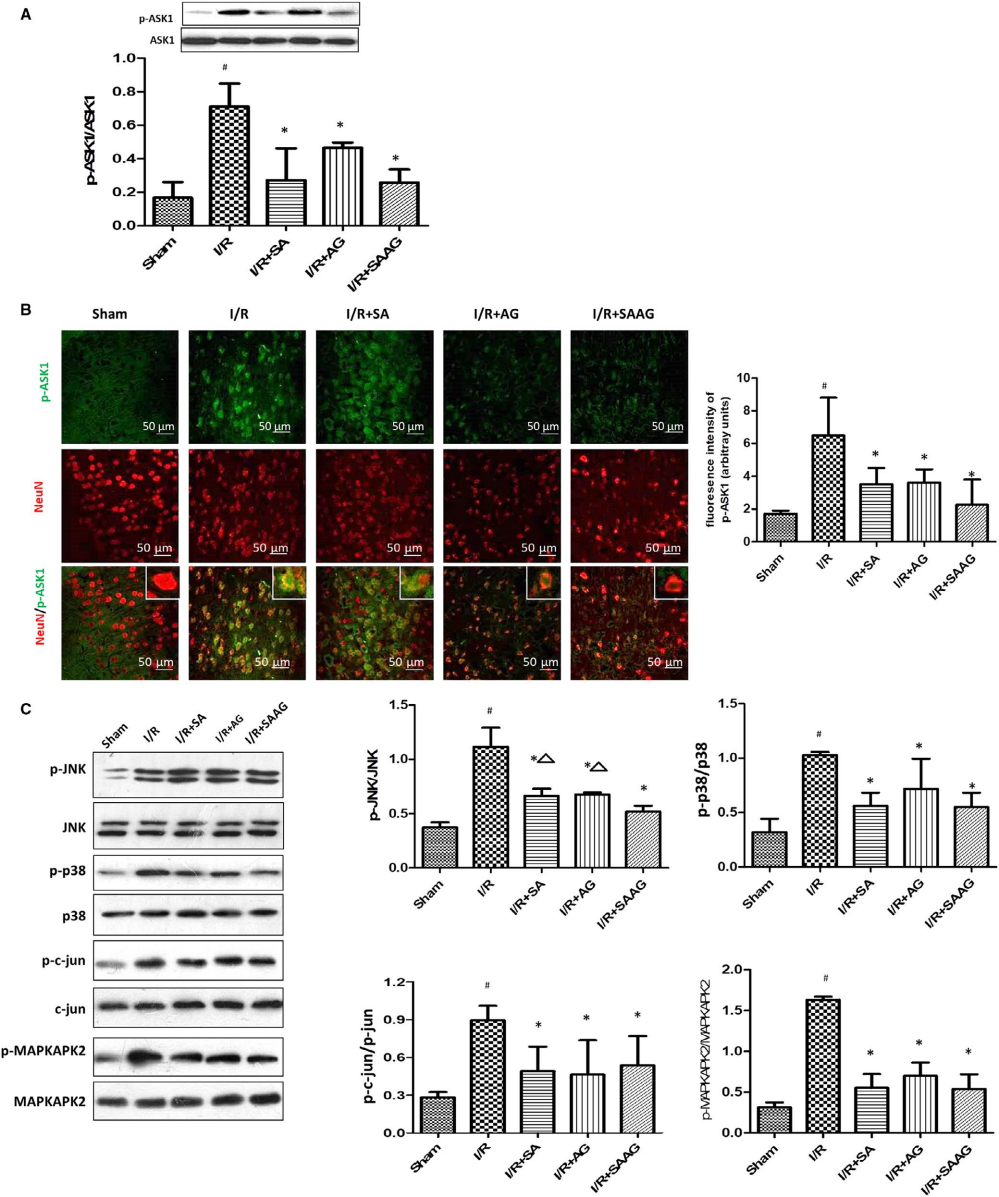
SAAG, SA and AG attenuated ASK1 activation and subsequent activation of the JNK and p38 signalling cascades. A, Western blotting of ASK1, p‐ASK1 and the quantitation of p‐ASK1/ASK1 (n = 3); B, Immunofluorescence staining for p‐ASK1 (green) and NeuN (red) and the quantitation of fluorescence intensity of p‐ASK1 (arbitrary units, n = 3‐5 animals per group); scale bar: 50 μm. C, Western blotting of JNK, p‐JNK, p38, p‐p38, p‐c‐Jun, c‐Jun, MAPKAPK2 and p‐MAPKAPK2 levels and their quantitation (n = 3). ^#^ *P* < .05 compared with the sham group, *P < .05 compared with the I/R group, and ^∆^ *P* < .05 compared with the I/R + SAAG group

Activation of ASK1 induces the phosphorylation of JNK and p38, resulting in neuronal apoptosis. The increased p‐ASK1/ASK1 level in I/R group was significantly decreased by SAAG, SA and AG (*P* < .05, Figure 4). ASK1‐dependent JNK and p38 signalling cascades were markedly activated in I/R group, as evidenced by the significantly increased phosphorylation of JNK, p38 and their downstream targets, MAPKAPK2 and c‐Jun (*P* < .05, Figure 4). In contrast, significant decreases in the phosphorylation of p38 and JNK and their respective downstream targets such as MAPKAPK2 and c‐Jun (*P* < .05) were observed following treatment of SAAG, SA and AG, indicating critical inhibition of the JNK and p38 cascades. Besides, the increased p‐ERK/ERK in I/R group was remarkably decreased by SAAG, SA and AG treatment (*P* < .05), which had no effect on the activation of AKT (Figure S1).

### 3.4 SAAG, SA and AG alleviated cell apoptosis and oxidative damage

**Figure 5:**
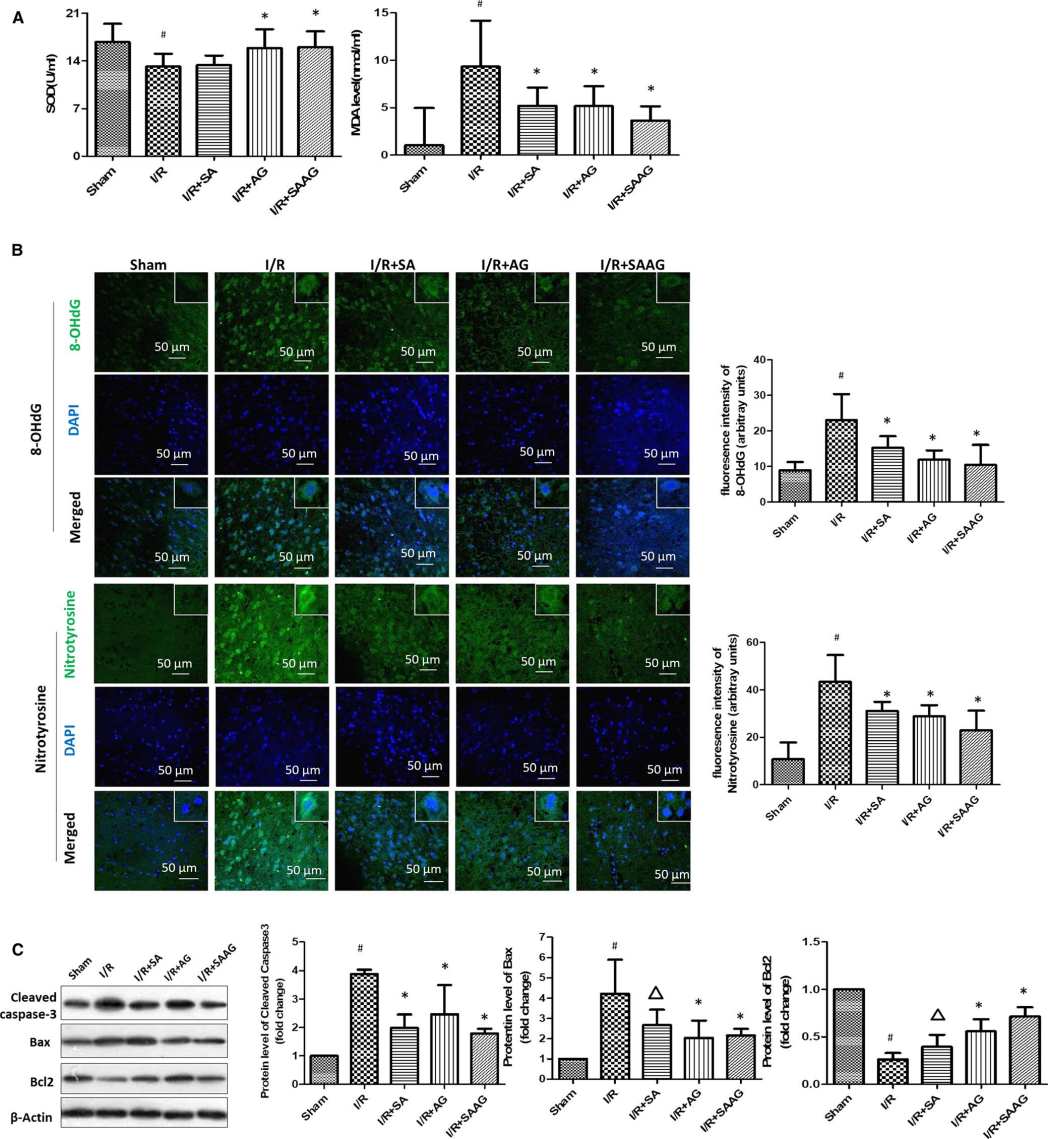
SAAG, SA and AG inhibited apoptosis and oxidative damage. A, The serum SOD activity and MDA content (n = 6). B, Immunofluorescence staining for 8‐OHdG (green), nitrotyrosine (green) and their quantitation of fluorescence intensity (arbitrary units, n = 3‐5 animals per group); nuclei (blue), scale bar: 50 μm. C, Western blotting of cleaved caspase‐3, Bax and Bcl2 levels and their quantitation (n = 3); ^#^ *P* < .05 compared with the sham group, *P < .05 compared with the I/R group, and ^∆^ *P* < .05 compared with the I/R + SAAG group

The decreased SOD activity in I/R group was increased by AG and SAAG (*P* < .05, Figure 4), and the MDA level was also obviously decreased by SA, AG and SAAG (*P* < .05). SAAG, SA and AG markedly decreased 8‐OHdG and nitrotyrosine in contrast to the I/R group (*P* < .05) (Figure 5B). Additionally, the AG and SAAG treatments significantly decreased the levels of cleaved caspase‐3 and Bax while increased Bcl2 levels (*P* < .05, Figure 5C). The SA treatment only significantly decreased the levels of cleaved caspase‐3.

### 3.5 Auranofin abolished the SAAG‐mediated protection

**Figure 6:**
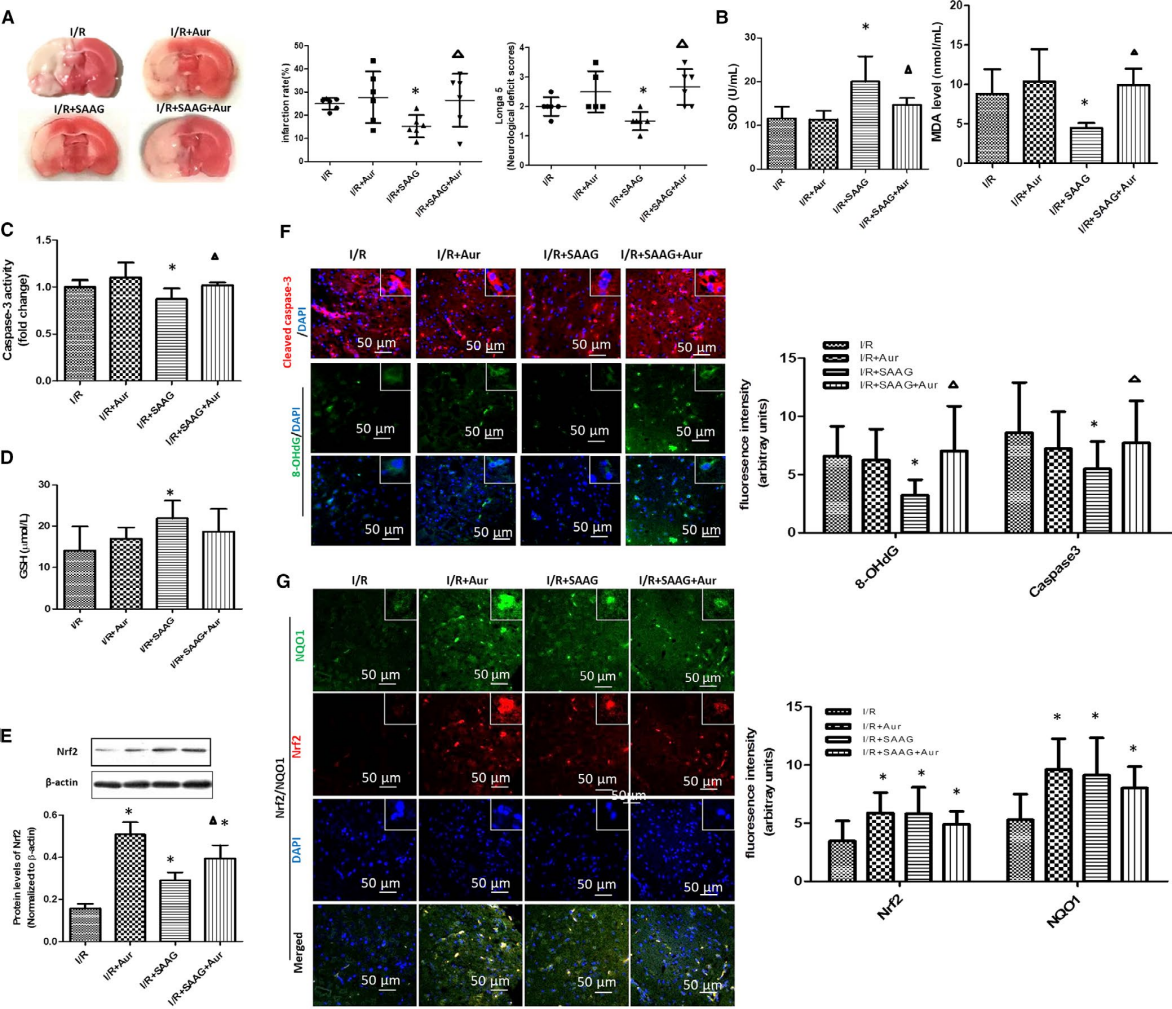
Inhibition of the Trx system reversed the protective effect of SAAG. A, Images of TTC staining, infarction rate and neurological deficient scores (n = 6). B, SOD activity and MDA content (n = 6). C, Caspase‐3 activity (n = 6), D, GSH levels (n = 6); E, Western blotting of Nrf2 and its quantitation (n = 3). F, Immunofluorescence staining for cleaved caspase‐3 (red), 8‐OHdG (green) and their quantitation of fluorescence intensity (arbitrary units, n = 3‐5 animals per group); nuclei (blue), scale bar: 50 μm. G, Immunofluorescence staining for Nrf2 (red), NQO1 (green) and their quantitation (arbitrary units, n = 3‐5 animals per group); nuclei (blue), scale bar: 50 μm. ^#^ *P* < .05 compared with the sham group, *P < .05 compared with the I/R group, and ^∆^ *P* < .05 compared with the I/R + SAAG group

Auranofin (Aur) alone increased the infarction rate and neurological scores after I/R treatment, but no differences was observed. Notably, the decreased infarction rate and neurological scores observed in I/R + SAAG group were further inhibited by Aur (*P* < .05, Figure 6A). Aur abolished the protective effect of SAAG on the antioxidant defences, as indicated by the significantly decreased SOD activity and increased MDA and 8‐OHdG levels as well as cell apoptosis, as evidenced by the increase of cleaved caspase‐3 staining and activity in I/R + SAAG+Aur group (*P* < .05). The GSH level observed in I/R + SAAG group was not affected by the Aur treatment, and the SAAG‐induced increase in Nrf2 levels was not repressed by Aur.

### 3.6 SAAG, SA and AG protected against H_2_O_2_‐induced neurotoxicity in PC12 cells

**Figure 7:**
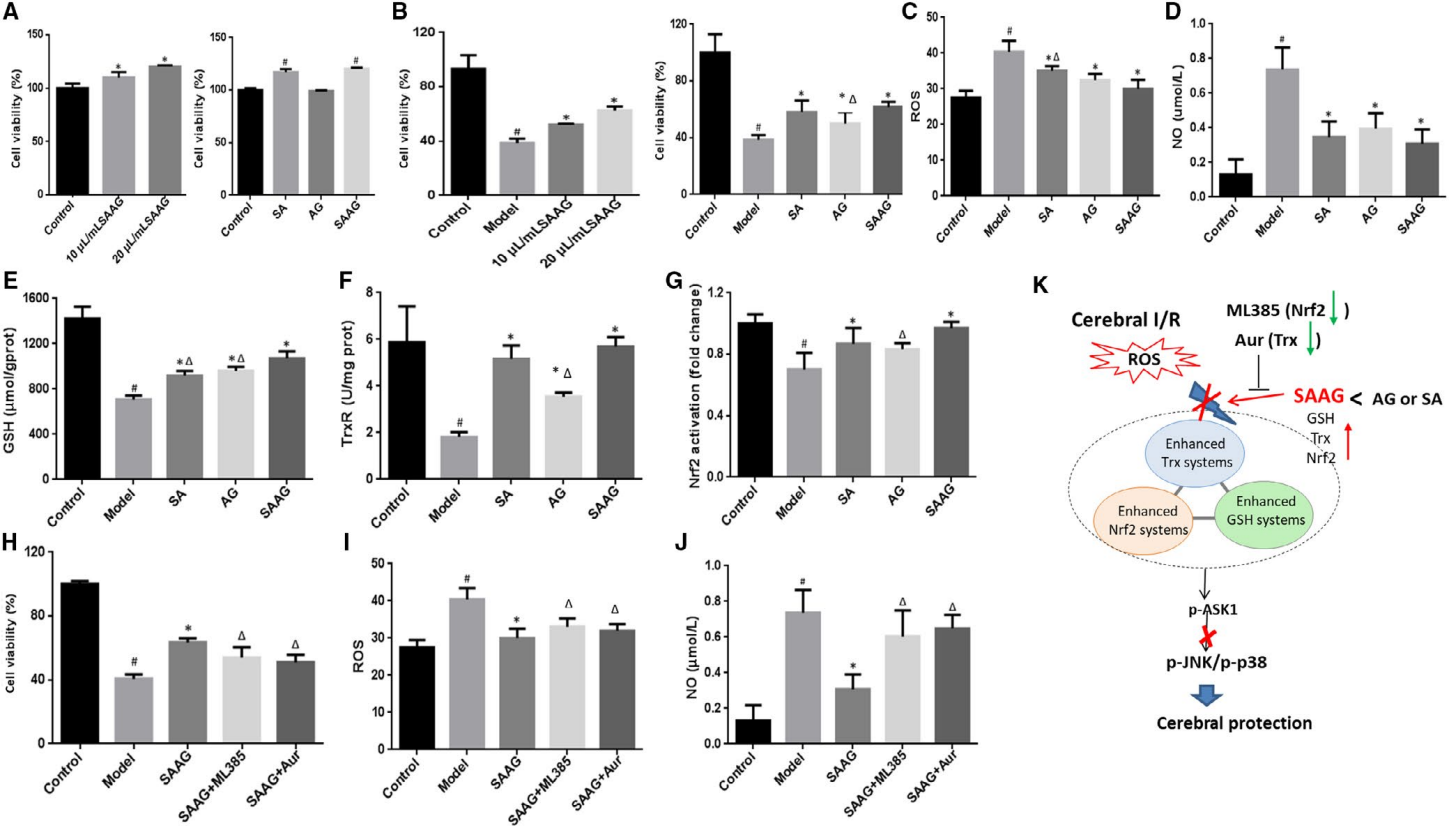
SAAG, SA and AG protected against H_2_O_2_‐induced neurotoxicity in PC12 cells; A, Viability of PC12 cells treated with SAAG, SA and AG (n = 5); B, Viability of PC12 pre‐treated with drugs and then followed with H_2_O_2_ treatment (n = 6); C, ROS level (n = 6); D, Nitric Oxide level (n = 4); E, GSH level (n = 4); F, TrxR activity (n = 3); G, Nuclear Nrf2 activation (n = 4); H, Cell viability by SAAG was abolished by Aur and ML385; (n = 6), I, Cellular ROS level enhanced by Aur and ML385 (n = 6); J, NO content increased by Aur and ML385 (n = 4); To reveal the role of SA and AG in SAAG, 0.6 mg/mL AG and 10 mg/mL SA was compared with 20 μL/mL SAAG in H_2_O_2_‐induced cell injury model, since every millilitre of SAAG contains 30 mg AG and 0.5 g SA according to its instructions; K, Schematic diagram illustrating that SAAG simultaneous enhanced the Trx, GSH and Nrf2 systems against cerebral I/R injury through ASK1‐JNK/p38 cascades. ^#^ *P* < .05 compared with the control group, *P < .05 compared with the model group, and ^∆^ *P* < .05 compared with the SAAG group

As indicated by Figure 7A, pre‐treatment of SAAG, SA and AG alone demonstrated no damage to PC12 cells. Because SAAG and its two components can directly scavenge ROS and reduce ROS‐mediated injury (Figure S2); thus, PC12 cells were pre‐treated with SAAG and its two components and then followed by H_2_O_2_ to avoid the disturbance of direct scavenging ROS. Safflower extract and aceglutamide and its two components demonstrated obvious protection against H_2_O_2_‐induced injury as indicated by increased cell viability, decreased cellular ROS and Nitric Oxide level when compared to model group. Specifically, higher cell viability than AG group and a lower ROS than SA group was observed in SAAG‐treated group (Figure 7B‐D).

In addition, an obvious repression in GSH level, TrxR activity and Nrf2 activation after H_2_O_2_, was significantly reversed by SAAG, SA and AG. Importantly, SAAG demonstrated a better improvement in GSH level, Nrf2 activation and TrxR activity than AG, while a higher GSH level than SA. These data indicated SAAG had a better enhancement in promoting triple antioxidant systems than either SA or AG. To reveal the role of Nrf2 system and Trx system in SAAG‐mediated protection, ML385 and Aur were used to inhibit Nrf2 and Trx, respectively. Notably, the improved cell viability by SAAG was significantly abolished by ML385 and Aur. Meanwhile, ML385 and Aur remarkably removed the inhibition of SAAG on cellular ROS level and Nitric Oxide level in H_2_O_2_‐induced PC12 model.

## 4 DISCUSSION

In cerebral I/R, ROS overproduction initiates extensive oxidative damage and results in cell death. In this study, we demonstrated that enhancement of GSH, Trx and Nrf2 systems by SAAG, demonstrated a better protection than its two components against cerebral ischaemia/reperfusion injury. The enhanced triple antioxidant systems inhibited the activation of ASK1, prevented its subsequent activation of the p38 and JNK signalling cascades, resulting in a reduction in oxidative damage and apoptosis (Figure 7K). Moreover, inhibiting Trx and Nrf2 system by Aur and ML385, respectively, abolished SAAG‐mediated protection, further highlighting the potential important role of the enhancing triple antioxidant systems in the protection of SAAG against cerebral ischaemia injury.

In this study, the enhancement of the GSH and Nrf2 systems by SAAG, SA and AG improved the cellular antioxidant capacity, resulting in decreased infarction rates and neurological scores. As the only antioxidant present at millimolar concentrations in the brain, GSH plays a vital role in modulating redox homoeostasis. Glutathione deficiency causes substantial oxidative stress and apoptosis^34^ and aggravates the cerebral infarct volume in subjects with cerebral I/R injury.^35^, ^36^, ^37^ Recently, GSH depletion in glial cells was reported to cause the release of TNF‐α and IL‐6, resulting in a 7‐fold increase in phospho‐p38 and phospho‐JNK and the induction of neuronal death.^38^ In contrast, the administration of GSH alleviates oxidative stress, reduces the infarct volume and prevents cell death.^16^ These findings confirmed a critical role for GSH in preventing cerebral injury, consistent with the results from our study. Additionally, the Nrf2 system plays a central role in modulating redox homoeostasis by binding to its antioxidant response elements (AREs) in the promoter regions of target genes in response to oxidative stress. Activation of Nrf2 exerts neuroprotective effects both in vivo and in vitro.^20^, ^39^ In contrast, Nrf2‐deficient mice are vulnerable to cerebral ischaemic injury.^40^ In our study, SAAG, SA and AG enhanced Nrf2 activation both in vitro and in vivo and inhibition of Nrf2 by ML385 deprived SAAG‐mediated protection in H_2_O_2_‐induced model, confirming the important role of enhancing Nrf2 in resisting I/R injury. Consistent with the findings from these studies, the enhancement of the GSH and Nrf2 systems by SAAG, SA and AG exerted an obvious protective effect on I/R damage.

Importantly, SAAG has a better enhanced effect on the Trx systems than either of its two compositions (Figure 3D). Trx plays a vital role in alleviating oxidative damage by passing electrons to Prxs and preventing the activation of ASK1.^18^, ^19^ Trx overexpression exerts a vital protective effect,^41^ whereas loss or oxidation of Trx induces ASK1 activation and leads to apoptosis.^42^, ^43^ As an antioxidant enzyme, Prx2 degrades peroxides such as H_2_O_2_ by obtaining electrons from Trx to protect neurons from ischaemia and oxidative injury.^44^, ^45^ In our study, SAAG demonstrated a better enhancement in promoting Trx systems than either SA or AG. Importantly, inhibiting Trx systems by Aur deprived the protective effect of SAAG both in vitro and in vivo. All these results demonstrated the vital role of enhancing Trx system in the protection of SAAG against cerebral I/R injury.

In this study, the SAAG enhanced the GSH, Nrf2 and Trx systems, allowing the three systems to complement and support each other, thereby enhancing their protection. More and more evidence have proved that though these three antioxidant systems work independently, they play vital roles in affecting each other. For example, the Trx system is an important regulator of the Nrf2‐Keap1 systems,^24^ and knockdown of Trx1 abolishes the protective effect of Nrf2 and produces a similar effect to Nrf2 inhibition on cerebral ischaemia.^23^ Consistent with our results that inhibition of the Trx systems with enhancement of Nrf2 system abolished the protective effect of SAAG. In addition, the Trx and GSH systems can compensate for each other in some situations, enhancing their capacity to resist oxidative damage.^19^, ^25^, ^46^ Importantly, GSH maintains the reduced form of Trx1 in the absence of TrxR1 activity.^25^ Additionally, buthionine sulfoximine (BSO)‐induced depletion of GSH results in Trx2 oxidation.^47^ The Trx system reduces GSSG to GSH, thereby regenerating GSH in GR‐deficient cells to alleviate cell damage.^48^ In our study, SAAG showed better enhancement of the GSH, Nrf2 and Trx systems at ameliorating the ischaemic damage in vivo and attenuating neurotoxicity in vitro than its two components, AG and SA. In contrast to the weak effect of SA and AG on the Trx systems, their combination (SAAG) showed strong improvement in the function of the Trx systems, probably due to the mutual complementarity and support of these three systems, and the enhanced Trx systems further stabilized the support between these systems, elevating the overall antioxidant capacity and thus providing better protection (Figure 7K). The enhanced Trx systems may be caused by the following three reasons. Specifically, SA and AG alone improved Trx level (not significant), and the combination of SA and AG (SAAG) may cause a cumulative increase of Trx to the level of significant difference. Additionally, Nrf2 stimulates the expression of a large number of antioxidant genes, including Txn1 (gene encoding Trx1 protein) and Txnrd1 (gene encoding TrxR1 protein) and a higher Nrf2 by SAAG may promote higher expression of Trx and TrxR.^49^ Finally, the significant increase in GSH level by SAAG may provide a backup effect for elevating Trx and TrxR levels.^25^

The enhancement of the Trx, GSH and Nrf2 systems work together to prevent the activation of ASK1 and subsequent p38‐ and JNK‐dependent apoptosis in cerebral I/R injury (Figure 7). Reactive oxygen species accumulation during I/R injury activates ASK1 and induces the activation of the JNK and p38 pathways to result in neuronal apoptosis.^50^, ^51^ Increased Trx levels prevent ASK1 activation and subsequently inhibit the activation of the downstream p38 and JNK pathways.^18^, ^19^ Additionally, the administration of glutathione disulphide markedly inhibits GSSG‐induced apoptosis and the ASK1/p38 MAPK signalling pathway.^52^ However, Nrf2 activation suppresses the p38 and JNK pathways and protects against cerebral ischaemic damage.^53^, ^54^ In our study, the enhancement of three systems induced by SAAG significantly decreased the phosphorylation of p38 and JNK and their downstream molecules, such as c‐Jun and MAPKAPK2 in I/R group, subsequently resulting in the noticeable inhibition of cell apoptosis. In addition, the activation of ERK1/2 inhibited by SAAG and its two components, may also affect cerebral I/R injury. However, the role of activation of ERK1/2 in cerebral ischaemia/reperfusion is controversial. Some researches demonstrated the protective effect of activating ERK1/2^55^ whereas other highlighted its inhibition benefited cerebral protection against I/R injury.^56^, ^57^

## 5 CONCLUSIONS

Taken together, enhanced triple antioxidant systems of Nrf2, GSH and Trx by SAAG demonstrated more effective in ameliorating oxidative damage after cerebral I/R than each of its two compositions. The simultaneous enhancement of triple antioxidant systems prevented the activation of ASK1 and subsequently inhibited the activation of p38 and JNK signalling cascades, preventing oxidative damage and apoptosis. This research provided the evidence for the necessity of combination drugs from the perspective of multiple antioxidant systems. Furthermore, this investigation offers clues and references for the study of combination drugs and inspires the discovery of novel treatments for ischaemic stroke.

## CONFLICT OF INTEREST

None.

## AUTHOR CONTRIBUTIONS

J. Zhang conducted animal study, immunofluorescence staining, conceived the study and wrote the manuscript; R. Zhou and C. Xiang performed cell culture, biochemical analysis and Western blotting; F. Fan, J. Gao, Y. Zhang and S. Tang performed the animal studies; H. Yang and H. Xu conceived the study; and all authors reviewed and approved the final manuscript.

## Supporting information

Appendix S1

## Data Availability

All data in this study can be obtained from the appropriate authors.
