# Supplementary material for: Enhanced thioredoxin, glutathione and Nrf2 antioxidant systems by safflower extract and aceglutamide attenuate cerebral ischaemia/reperfusion injury
Source: J Cell Mol Med. 2020 Apr 7;24(9):4967–80. doi: 10.1111/jcmm.15099 (PMC7205826; doi:10.1111/jcmm.15099)
Supplement: Supplementary file 1 — Appendix S1 [file JCMM-24-4967-s001.docx]

**Supplementary materials**

**Materials and methods**

**Drugs and antibodies**

The AG (batch number: 1705002) was purchased from Shanxi West Yue Pharmaceutical Co., Ltd, and the SA (batch number: 20171010) was obtained from Xinjiang Daze Honghua Pharmaceutical Co., Ltd. Safflower extract and aceglutamide injection (SAAG) is a standardized medicine product recorded in the Chinese Pharmacopeia and is widely used for various cerebral diseases, such as clinical cerebral insufficient blood supply and cerebral thrombosis and embolism. The production of SAAG injection was strictly performed according to the Chinese national standard [No. WS-10001-(HD-1506) -2004] published by the Chinese Pharmacopoeia Commission. According to the SAAG instructions, every milliliter of SAAG contains 30 mg AG and 0.5 g SA. SAAG as a combination of SA and AG, and the composition of SAAG was identified by high-performance liquid chromatography (HPLC); seven effective components, including aceglutamide, hydroxysafflower yellow A, uridine, adenosine, guanosine, syringing, and anhydrosafflor yellow B, were identified and quantified when compared with the reference substances.

The antibodies used in this research were as follows: anti-Nrf2 (ab137550), anti-NQO1 (ab28947), anti-thioredoxin/TRX (ab109385), anti-peroxiredoxin 2 (ab109367), anti-ASK1 (CST8662S), anti-p-ASK1 (bs-3031R), anti-phospho-c-Jun (Ser63) (CST9261), anti-c-Jun (ab32137), anti-MAPKAPK-2 (ab32567), anti-p-MAPKAPK-2(YP0577), anti-p38 MAPK (8690T), anti-JNK1/2 (sc-137019), Anti-JNK1+JNK2 (phospho T183 + Y185)(ab131499), anti-p38 (phospho T180 + Y182) (ab195049), anti-cleaved caspase-3 (CST, 9664S), anti-Bax (ab182733) and anti-Bcl2 (ab196495), anti-glutathione reductase (sc-133136), anti-GCLM (ab124827), anti-nitrotyrosine (sc-101358), and anti-8-OHdG (sc-66036).

**MCAO surgery and drug administration**

Rats were anesthetized with sodium pentobarbital (45 mg/kg, intraperitoneal injection, i.p.) and were subjected to MCAO surgery and reperfusion. Briefly, a 4-0 nylon thread with a round side was inserted into the right common carotid artery and deep into the origin of the left middle cerebral artery to occlude the artery. After 90 minutes, the occluded thread was removed to allow blood flow. Those rats whose brain sections showed no ischemia were excluded from the experimental group. Then, the rats were placed back in their cages and allowed free access to food and water. The rats Sham group received the same surgery except for insertion of the thread. According to equivalent dose conversion according to clinical dose and the previous publication [1], our previous research determined that 2.5 ml/kg of SAAG was appropriate dose for cerebral ischemic I/R [2]. After finding the appropriate dose of SAAG, then the doses of its two components SA and AG were determined according to the composition of SAAG. The rats were randomly assigned into the following groups: Sham group(treated with physiological saline, i.p.), I/R group (subjected to I/R and treated with physiological saline, i.p.), I/R+AG (subjected to I/R and treated with 75mg/kg AG, i.p.), I/R+SA group (subjected to I/R and treated with 1.25 g/kg SA, i.p.), I/R+SAAG group (subjected to I/R and treated with 2.5 ml/kg SAAG, i.p.), and I/R+ginaton group (subjected to I/R and treated with 8 ml/kg ginaton; positive control, i.p.). All of the groups were derived from a random number table. Oxidative stress markers such as plasma lipid peroxides and urine 8-isoprostanes are the highest within 24 h after the ischemic stroke attack [3] and an increase of plasma level of F2-isoPs (an oxidative marker,) appeared at as early as 8h after stroke onset [4], indicating targeting oxidative stress should be performed at the early stage of cerebral I/R. Thus, we designed the pre-experiments that administration of drugs immediately before reperfusion and then once a day by intraperitoneal injection following I/R. The rats were sacrificed until a significance of the neurological deficient scores was observed in I/R+SAAG group compared to I/R group. After determining the dose of SA and SAAG, the dose of SA was calculated according to the SAAG instruction, since every milliliter of SAAG contained 30 mg AG and 0.5 g SA. All drugs were delivered by intraperitoneal (i.p.) injection immediately before and 24 h after reperfusion, respectively. The rats were sacrificed 48 h after the I/R, and serum and brain tissue were harvested for the following experiments.

**Western blotting**

The protein samples were quantified by a Beyotime Protein Assay Kit (Nanjing, China). After denaturation, the proteins were separated by electrophoresis on polyacrylamide gels, followed by transfer of the proteins to polyvinylidene difluoride membranes, which were blocked with bovine serum albumin (BSA). Then, the membranes were incubated with anti-ERK1/2, (CST, 4695T), anti-Erk1/2 (Thr202/Tyr204) (CST, 9101S) anti-AKT (proteintech, 10176-2-AP), anti-p-AKT(phosphor-S473) (proteintech, 66444-1-lg) followed by incubation with HRP-conjugated secondary antibody (Jackson 111-035-003). Using an enhanced chemiluminescence plus detection system (Pierce Biotechnology, Rockford, IL, USA), the protein signals on the membranes was quantified by scanning densitometry with image analysis software (Science Lab 2005 Image Gauge; Tokyo, Japan).

**Direct scavenging ROS and H2O2**

The scavenging effect of SAAG, SA and AG on ROS and H_2_O_2_ was evaluated by incubating SAAG, SA and SA with ROS containing medium and H_2_O_2_ solution for 3 hour, respectively. After that, the ROS and H_2_O_2_ level was detected by using DCF ROS/RNS Assay (ab238535) according to its instruction. The fluorescence was detected with a microplate reader (Molecular Devices, USA) at 480 nm excitation/530 nm emission. As for the effect of scavenging ROS, culture medium was harvested after the induction of PC12 cells with 700μM H_2_O_2_ for 3 hour, which was used as ROS containing medium.

**Results**

As indicated in Figure S1, the MCAO-induced I/R injury caused a significant in increase in p-ERK/ERK, whereas it was remarkably decreased by SAAG, SA and AG. In addition, SAAG and its two components had no effect on the activation of AKT.


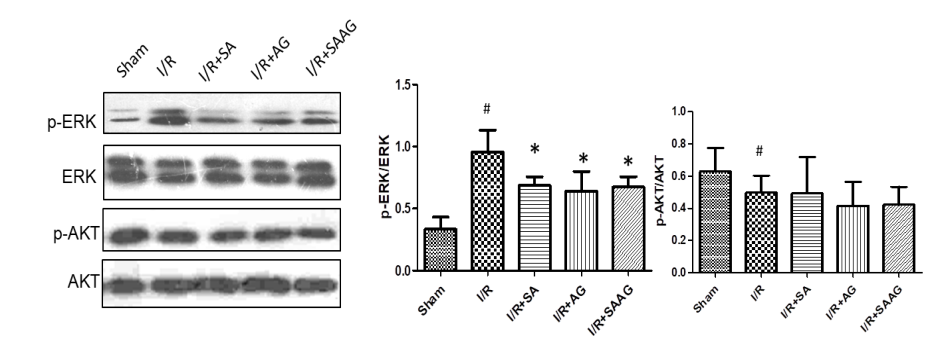


Figure S1. Western blotting results of ERK1/2, p-ERK1/2, AKT, p-AKT in MCAO-induced I/R model and its quantitation, (n=3) ^#^P<0.05 compared with the sham group, *P<0.05 compared with the I/R group.

As indicated by Figure S2, SAAG, SA and AG at higher concentration had an obvious scavenging effect on H_2_O_2_ directly. As for ROS, SAAG and AG had an effect in decreasing ROS level. These data indicated that SAAG, SA and AG had a direct scavenging effect on H_2_O_2_ and ROS levels.


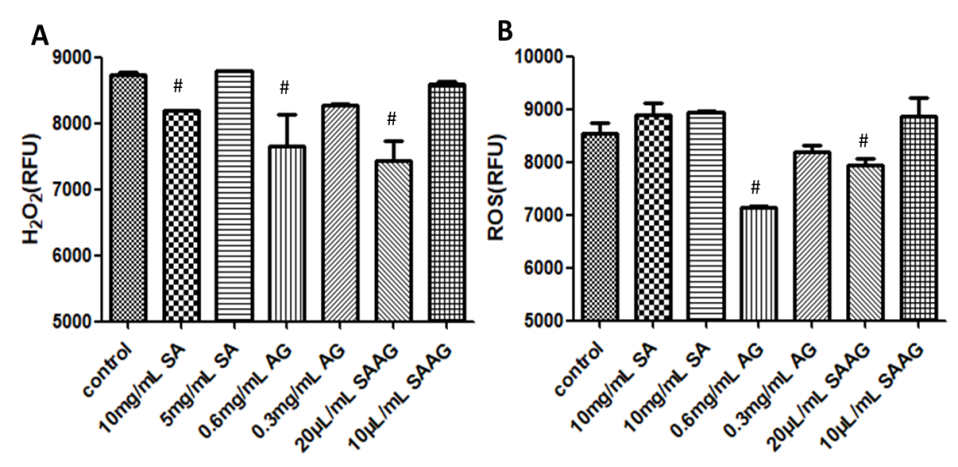


Figure S2. The direct scavenging effect of SAAG, SA and AG on H_2_O_2_ and ROS; (A) H2O2 (n=3); (B) ROS (n=3); ^#^P<0.05 compared with the control group.

**Reference**

[1]. **Ai J, Wan H, Shu M, *et al*.** Guhong injection protects against focal cerebral ischemia–reperfusion injury via anti-inflammatory effects in rats*.* *Archives of Pharmacal Research*. 2017; 40:610-22.

[2]. **Fan FF, Chen SM, Yang HJ, *et al*.** The investigation of molecular mechanism of Guhong injection against cerebral ischemia-reperfusion injury in network pharmcology approach*.* *Complex System and Complexity Science*. 2018; 15:11-7.

[3]. **Žitňanová I, Šiarnik P, Kollár B, *et al*.** Oxidative Stress Markers and Their Dynamic Changes in Patients after Acute Ischemic Stroke*.* *Oxidative Medicine & Cellular Longevity*. 2016; 2016(ID 9761697):7.

[4]. **Kelly PJ, Morrow JD, Mingming N, *et al*.** Oxidative stress and matrix metalloproteinase-9 in acute ischemic stroke: the Biomarker Evaluation for Antioxidant Therapies in Stroke (BEAT-Stroke) study*.* *Stroke*. 2008; 39:100-4.
